# What can clinical leaders contribute to the governance of integrated care systems?

**DOI:** 10.1136/leader-2022-000709

**Published:** 2023-03-08

**Authors:** Justin Waring, Simon Bishop, Georgia Black, Jenelle Clarke, Bridget Roe

**Affiliations:** 1 Health Services Management Centre, University of Birmingham, Birmingham, UK; 2 Centre for Health Innovation, Leadership and Learning, University of Nottingham, Nottingham, UK; 3 Wolfson Institute, Queen Mary, University of London, London, UK; 4 Social Policy, Sociology and Social Research, University of Kent, Canterbury, UK

**Keywords:** clinical leadership, integration, health system

## Abstract

**Background:**

Integrated care systems present enduring governance challenges associated with fostering interorganisational collaboration.

**Aim:**

To understand how clinical leaders can make a distinct contribution to the governance and system leadership of integrated care systems.

**Methods:**

A qualitative interview study carried out between 2018 and 2019 with 24 clinical leaders, and a further 47 non-clinical leaders, involved in the governance of three Sustainability and Transformation Partnership in the English National Health Service.

**Results:**

Clinical leaders were found to make four distinct contributions: (1) making analytical insights into integration strategies that ensured their relevance and quality to clinical communities; (2) representing the views of clinicians in system decision-making thereby enhancing the legitimacy of change; (3) translation and communication activities to articulate integration strategies in favourable ways and ensure clinical engagement; and (4) relational work in the form of brokering and building connections and mediating conflict between multiple stakeholders. These activities varied across the levels of system governance and at different stages in the processes of change.

**Conclusions:**

Clinical leaders can make a distinct contribution to the governance and leadership of integrated care systems based on their clinical expertise, membership professional networks, reputation and formal authority.

**WHAT IS ALREADY KNOWN ON THIS TOPIC**

- System leadership is integral to the governance of integrated care systems where multiple actors steer system organisations towards collaborative working.

**WHAT THIS STUDY ADDS**

- This study shows the distinct contribution clinical leaders positioned at different levels can make to system leadership for integrated care systems.

**HOW THIS STUDY MIGHT AFFECT RESEARCH, PRACTICE OR POLICY**

- This study identifies key clinical leadership activities that need more explicit recognition by those involved in system leadership and which might inform leadership development.

## Introduction

 There is a long history of calls to integrate health and social care services.^1^ This has recently centred on the creation of regional integrated care systems, in which established primary, secondary, community and social care services within a given locality are reconfigured to better meet the health needs of local communities.^2^ They are advocated on the grounds of improved access, quality and joined-up care, tackling engrained population health issues, delivering operational efficiencies and reducing demand on episodic acute care in favour of more continuous and patient-centred primary and community care.

In the last decade, there has been a gradual shift towards integrated care systems in the English health and social care system.^1^ This has included Integrated Care Pioneers, New Care Models, Sustainability and Transformation Plans, later Partnerships (STPs) and, now, Integrated Care Systems (ICSs). In 2014, the *Five Year Forward View*^2^ introduced STPs, the empirical focus for this paper, as a partnership arrangement in which regional health and social care agencies collaborate to plan the delivery of integrated care services. In 2016, 44 STPs were introduced across the English National Health Service (NHS) to address well-documented problems in primary care, prevention, early intervention, mental health, productivity and workforce development. The *NHS Long Term Plan*^3^ of 2019 proposed that STPs evolve to become independent ICSs and, following the *Health and Social Care Act* of 2022, 42 ICSs were introduced across England to plan and coordinate integrated place-based care at the regional, place-based and neighbourhood levels. ICSs are described as an advanced version of STPs. Unlike STPs which were largely based on voluntary joint working with limited budgetary responsibilities, ICS have statutory powers, through regional Integrated Care Boards, to both devise plans to meet local health and care needs and to allocate NHS resources to meet these needs, thereby replacing the existing commissioning arrangements.

There remain significant questions about the governance of integrated care systems.^4 5^ These centre on how best to steer interorganisational collaboration and resources sharing in a context where there has been a history of competition, where those responsible for leading change can lack formal authority and where prevailing governance arrangements exert influence over constituent health and social care organisations. Systems working is often premised on the capacity of leaders to foster shared commitment to integration through shaping cultures, influencing partners and mediating conflict.^6^ According to Edmonstone,^7^ there is a need for shared multi-agency leadership that transcends professional, organisational and sectoral silos and fosters horizontal collaboration. Tweed *et al*^8^ further highlight the importance of fostering allegiances between stakeholders through (1) relational work and brokering connections; (2) framing and translating a shared vision and (3) engaging in practical activities in developing and learning from projects. A recent review of the literature found that despite mounting evidence of the role of leaders in shaping governance arrangements, more research is needed on the competencies and contributions of network leadership in integrated care systems.^6^

There is an expectation that clinical leaders can play a pivotal role in the leadership of integrated care systems.^2 3^ Clinical leadership is conceptualised in many ways. We use the term clinical leadership to describe a health and care professional who is involved, either formally or informally, in the processes of shaping change by contributing, for example, to strategic development, social influence, communication and empowerment. This acknowledges that the processes of leadership can be shared or distributed whereby many clinicians and non-clinicians contribute to the process of change, but where clinicians can make a distinct contribution by virtue of their professional expertise, relationships and standing.

Past research shows that clinical leaders can make important contributions to service change. Fitzgerald *et al*^9^ show how distributed multitiered leadership, especially by hybrid clinical leaders, can facilitate improvement within healthcare organisations through the framing of strategy to stakeholders, translating policies, engaging staff and maintaining momentum. Jones and Fulop’s^10^ study of hospital-board Medical Directors further describes the contribution of these clinical leaders to the governance of quality within and beyond their organisations. This includes translational work of interpreting data and providing analytical insight; diplomatic work of managing conflict and maintaining relationships and repair work of rebuilding relationships that have broken down. Furthermore, research on the introduction of regional-level care networks further shows that clinical leaders are well-placed to shape strategy development, build consensus and mediate conflict among professional communities because of their clinical expertise, developed connections within and across clinical communities and their formal and informal authority with stakeholders.^11^ These qualities suggest clinical leaders can make a significant contribution to the governance of integrated care systems.^12^ However, the contribution of clinical leaders remains unclear, especially as integrated care systems involve changed governance arrangements that place growing emphasis on interorganisational collaboration that potentially creates new forums for leadership action outside individual organisations.

To better understand the contribution of clinical leaders to the governance of integrated care systems, the study reported in this paper was informed by three relevant leadership theories. First, network leadership theory suggests the tasks of coordinating interorganisational networks are distinct from traditional forms of intraorganisational leadership. McGuire^13^ elaborates these as: (1) the ability to identify and engage stakeholders; (2) to facilitate agreement among stakeholders about the purpose, norms and rules of integrated working; (3) to foster and sustain commitment; and (4) to create an environment that facilitates resource sharing. Second, and with consideration to the idea that integrated care systems might involve more collaborative consensus-driven decision-making,^12^ Ansell and Gash^14^ highlight the importance of ‘facilitative leadership’ in collaborative governance regimes, especially in bringing stakeholders to the table, setting the ground roles and steering collaboration, mediating interactions, building trust, empowering others, exploring mutual gains and acting as honest brokers. They elaborate three leadership approaches.^15^ ‘Stewards’ facilitate collaboration and maintain the integrity of relationships by establishing the inclusivity and transparency of collaborative processes; ‘mediators’ arbitrate between stakeholders, mediate disputes and foster shared understanding; and ‘catalysts’ mobilise collaboration by identifying and focusing on shared values and nurturing mutually beneficial connections. Third, Senge *et al*^16^ outline a concept of ‘systems leadership’ that accounts for the potential for multiple agencies to come together in collaborative problem-solving. This is associated with three capabilities: (1) the ability to see the big picture and foster shared understanding; (2) enabling reflective and generative conversations so that partners can better learn about themselves and others; and (3) nurturing a proactive and inspirational form of collective action. These ideas provided a heuristic guide for data collection and analysis where the purpose of this study was to investigate the distinct contribution of clinical leaders to the leadership of integrated care systems.

## Method

### Recruitment and sample

The study was undertaken in the English NHS between 2018 and 2021, focusing on the governance of three STPs. Following a desk review of all 44 STPs, three sites were selected on the basis of differences in the number of healthcare and social care organisations and local demographics. The study investigated the contribution of clinical leaders at multiple levels of STP governance. Although integrated care systems are promoted as fostering inter-agency collaboration, research shows that such networked forms of governance can also introduce new hierarchies.^11^ In the case of the STPs, regional decision-making groups were positioned, at least symbolically, above individual care provider organisations to lead interorganisational working, and each STP was designed to include multiple levels of leadership through which strategies and plans for system change were formulated, prioritised and implemented. This included executive leadership (concerned with overall strategy, planning and coordination), thematic leadership (concerned with coordinating and overseeing programmes of system change in areas such as maternity or mental health) and project leadership (concerned with devising and delivering collaborative system change initiatives).

For our study, clinical leaders were defined as people with a primarily clinical background and/or role who also held a leadership role within the integrated care system at one of these three levels. Study participants were recruited on the basis of their involvement in STP governance and identified by reviewing public documentation and observing STP meetings. In total, 25 clinical leaders were recruited from across the three STPs (see table 1), together with a further 47 non-clinical leaders involved in STP governance, such as Chairpersons, Managing Directors, Project Managers, Workforce Managers, Finance Managers and other administrators. It was important to include such groups in the study to allow for reflections on the activities and behaviours of clinical leaders from the position of their non-clinical counterparts.

**Table 1:** Sample of participants with designated clinical leadership role

	STP A	STP B	STP C
Executive	STP Director (GP). Medical Director.	Medical Director.	Medical Director.*
Thematic	Medical Lead for Family Medicine. Medical Lead for Acute Medicine. Pharmacy Lead for Medicines.	Medical Lead for Mental Health.* Medical Lead for Urgent Care. GP Lead for Primary Care.	Medical Director for Community Services. GP Lead for Primary Care.
Project	Midwifery Lead. Obstetrics Lead. Pharmacy Practice Lead. Community Pharmacy Lead. GP Representative.	Trauma Lead (Medical).* Rehabilitation Lead (Nursing).* Community Mental Health Lead (Medical).* GP Network Representatives (3).	Community Nursing Lead. Cancer Services Lead (Nursing).
Total	10	10	5

* iIndicates interviewed more than once.

GP, General Practitioner; STP, Sustainability and Transformation Plan.

### Data collection

All 25 clinical leaders participated in semi-structured interviews that investigated the history and governance of each STP and the role of clinical leaders within these arrangements. Interviews lasted on average 1 hour, and were recorded for transcription and analysis. Five clinical leaders participated in follow-up interviews to trace the changes in STP leadership, all within 6 months of the initial interviews. All participants gave written consent to take part in the study. Data collection at the third site was limited due to the COVID-19 pandemic, including limits on recruitment due to resource pressures and limits of direct observations.

Non-participant observations were carried out with executive meetings, thematic committee meetings and project team meetings. This enabled direct observation of clinical leaders’ activities and contributions. Across the three STPs, 28 meetings were observed over 49 hours (STP A: 22 hours, STP B: 22 hours and STP C: 5 hours). As part of these observations, many of the recruited clinical leaders participated in informal field interviews to explain events and clarify observations. Observations were written up in field journals followed by electronic summary reports.

### Data analysis

Interpretative data analysis was undertaken through deliberative discussion and recurrent validation by the authors. An initial sample of transcriptions was reviewed by all authors to identify relevant themes, which informed subsequent coding. Systematic coding was undertaken by (Waring and Roe) which involved line-by-line review of data to code sections of data. Codes were reviewed by all authors for their descriptive accuracy, internal consistency and conceptual connections. Second-order codes were identified based on the connections of initial coding, which were further grouped into broader themes. The final stage involved relating these themes back to leadership theories as a basis of further thematic refinement and interpretation (see online supplemental file 1). The study findings were also presented and deliberated with healthcare leaders in four workshops with a focus on the practical lessons for practice.

## Results

### The clinical leaders

The 24 clinical leaders were drawn from a number of clinical backgrounds (medicine, nursing, pharmacy, occupational therapy), with varying degrees of leadership experiences, and different roles across the levels of system governance. The three STP medical directors had primary clinical careers in acute hospital medicine, but also extensive experience as leaders of clinical departments, medical directors of their respective hospital organisations. One of the STP chief executives also had a clinical background as a General Practitioner (GP) and had previously acted as medical lead within local commissioning bodies before moving into a largely management role. At the thematic level, eight clinical leaders also had extensive prior experience of leadership within their respective organisation and had acquired new system leadership roles for system transformation within given specialist clinical areas, for example, family medicine, mental health or primary care. At the project level, clinical leaders had less senior leadership experience, but all had existing clinical leadership responsibilities within existing services, such as team and departmental leaders, were highly regarded figureheads and had experience in supporting new models of service delivery. Given the differences in their position within the STP governance arrangements, there were different views about both the importance of system change and the ways in which it might be operationalised, with some focusing more on the strategic integration of resources and services and others on the everyday delivery of care. Such differences were further reflected in the types of contributions clinical leaders made to their integrated care system.

### Analytical insight

At all levels of STP governance, clinical leaders provided analytical insight into decision-making, which made integration strategies relevant and realistic from the perspective of multiple stakeholders. At senior levels, this involved informing the overarching integration strategy by, for example, interpreting data on population health needs or presenting the evidence for clinical priorities. At the thematic level, it involved providing authoritative guidance on service priorities, reflective of the clinical leader’s background. At the project-level, it involved devising tangible service transformation plans, reflecting their developed clinical expertise within a given service, and their familiarity of the day-to-day realities of service delivery.

This analytical insight took a number of forms. First, clinical leaders were skilled at reviewing and presenting clinical evidence and service data, and most had a developed understanding of research in particular specialist areas. Second, more experienced clinical leaders had a developed appreciation of the underlying tensions and historic contingencies that had previously complicated integration initiatives, and so could inform planning around potential points of disagreement. Third, and linked to the above, clinical leaders were influential in determining feasible or workable priorities given their understanding of local professional cultures, which also helped decision-makers avoid possible pitfalls. Through their analytical capabilities, clinical leaders therefore helped collaborative decision-makers formulate more realistic and relevant integration plans.

### Representation

Clinical leaders described their contribution to STP governance as representing the views of professional colleagues in different decision-making arenas, which in turn enhanced the legitimacy of integration strategies across multiple stakeholders. Although senior leaders took a broader ‘systems views’, their ability to understand and articulate the clinical realities of frontline healthcare professionals, both from their own discipline and others, was important to their role. This seemed important given that decision-making occurred outside of established organisational or clinical settings and, in some sense, was more remote from frontline services. This contribution was therefore more pronounced with those leading thematic areas and transformation projects, whose continued involvement in the delivery of frontline care meant that representing the views of professional colleagues into decision-making was central to their role.

The representational role took a number of forms. First, was the ability to ensure clinical views or ‘voice’ was included in decision-making, thereby ensuring that decisions had greater sense of legitimacy with professional stakeholders. Second, it involved offering critical challenges during decision-making, especially for ensuring clinical concerns were equal to managerial or financial priorities. Third, and linking the former, clinical representation contributed to the ensuring wider professional engagement and buy-in to the STP programmes.

### Translating and communicating

A prominent contribution of clinical leaders was their ability to not only represent the views of professional stakeholders but also to favourably translate and communicate integration plans in ways that promoted professional engagement. At senior levels, this focused on the broad vision and mission of the STPs, at the thematic level it centred on explaining the prioritisation and selection of transformation projects to professional communities, and at the project level it involved setting out the specific purposes, approaches and measures for a given system transformation initiative.

These activities took a number of forms. First, clinical leaders at executive, thematic and project levels were integral to the translation of evidence, policy and proposals between stakeholder communities; especially between clinical and non-clinical groups. This attended to the appropriateness of language, summarising complex evidence, or setting out the rationale for system change. Second, leaders were effective at framing change in ways that aligned with the prevailing concerns of different stakeholders, especially through the use of evidence or focusing on the shared concerns to justify a given initiative. This included appreciation of the issues to downplay to avoid unwelcome responses as well as the issues to emphasise to foster engagement. A common idea was that leaders could help clinicians see the ‘big picture’ as a basis for collaborative involvement. Third, communication activities were often concerned with sustaining engagement and so, overtime, they involved ‘keeping the story going’ with a focus on telling a shared narrative of collaboration with achievements attributed to multiple stakeholders.

### Relational activities

Clinical leaders performed relational activities that contributed to creating and sustaining collaboration across regional partners, especially professional groups. Across the levels of STP governance, these activities took slightly different forms according to scale and seniority. For example, senior clinical leaders were often engaged in securing the involvement of a relatively small group of senior managers or leaders of other care organisations, whereas those involved in thematic or project activities focus on a broad range of relationships at less senior levels.

Relational work took a number of forms. First, clinical leaders described working to broker and build connections between multiple stakeholders, often with the intention of securing engagement in collaborative activities. In most cases, these connections were nurtured to foster shared understanding and mutual benefit. Second, and building on the first, clinical leaders talked about building self-sustaining alliances and networks among system actors, which involved creating a critical mass of like-minded people interested in changing established ways of working. Third, relational activities often had an empowering but also delegatory function, in that groups were formed and encouraged to take forward the integration plans as set-out by clinical leaders, but in some cases, this involved more directive task allocation. This was especially evident with those involved in theme leadership where local groupings of service providers had delegated responsibility for devising and delivering on transformation projects. Finally, clinical leaders had an important role in mediating conflict, which for many was based on understanding the history of tensions in the region and their developed appreciation of the interests that set groups in opposition. Through this insight, they described themselves as avoiding or reducing conflict, and knowing the inducements that could placate opponents and foster collaboration.

## Discussion

This study finds that clinical leaders make a number of interconnected contributions to the governance of integrated care systems that address many of the tasks described in the literature related, for example, relationship building, framing a shared vision, engaging stakeholders and fostering commitment.^13 15 16^ Each thematic contribution (analytical, representational, translational and communication, and relational) comprises a number of activities and it is through the combination of these activities that clinical leaders contribute to the different stages and tiers of system governance. These activities have a primary or direct effect and in combination they produce secondary or latent effects.

Clinical leaders’ analytical insight about, for example, historical trends or performance data has the primary effect of ensuring strategic plans are relevant and realistic to the needs of local services, which has the consequent effect of promoting stakeholder engagement and commitment to change. Representing the views of professional colleagues ensures integrattion plans reflect the interests and priorities of these stakeholders which in turn enhances the perceived legitimacy of change (and also the legitimacy of the clinical leader). Translational and communication activities function in a linked way, whereby leaders convert and then share knowledge across syntactic and semantic boundaries thus making change meaningful to stakeholders, which then fosters a shared vision and commitment for change. Relational activities connect stakeholders in amicable and reciprocal ways, which facilitates the sharing of resources and sustained collaboration. These activities function in overlapping and mutually reinforcing ways to produce a range of higher-order contributions in the form of shared vision, legitimacy for change, stakeholder engagement and commitments and the sharing of resources.

**Figure 1:**
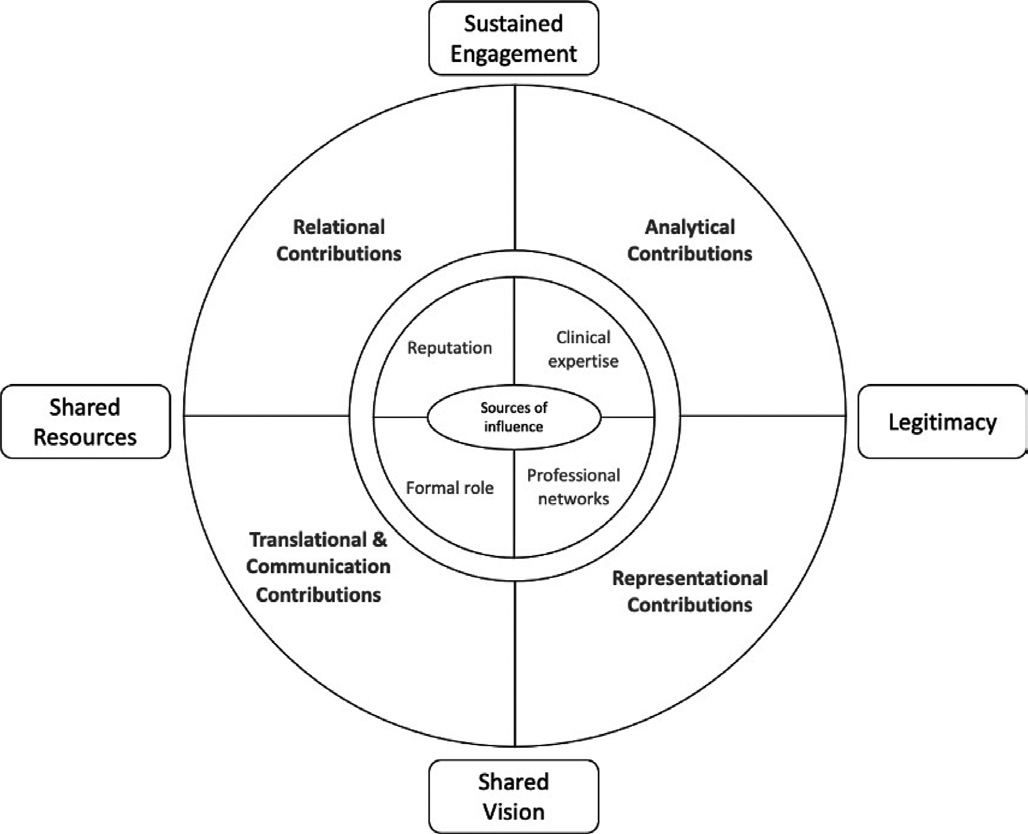
Contribution of clinical leaders to system leadership.

The study does not assume that other system leaders, without clinical backgrounds, cannot make these contributions, but it does suggest that clinical leaders are well-placed to undertake these activities. Furthermore, they are especially well-placed to secure the engagement and commitment of professional groups who can be conservative about change. Developing this idea, clinical leaders draw on particular sources of influence and power that establish their distinct position relative to other system actors.^17^ Specifically, they have extensive clinical expertise and experience often with subspecialist credentials that underscores their analytical insights; they have developed networks within and across professional communities that facilitates their translational and communication activities; and they have both formal authority based on designated leadership role and informal influence based on professional standing, which together support their relational and representational activities. Figure 1 illustrates clinical leaders’ underlying sources of influence (expertise, networks, formal authority, informal influence) that combine to enable their primary contributions to system leadership (analytical insight, representation, translation and communication and relational) and which further combine to produce secondary effects in system governance (shared vision, legitimacy, engagement, resource sharing).

What does our study contribute to the existing research and theory? As outlined above there are calls for clinical leaders to contribute to system leadership by, for example, brokering connections, framing and translating vision, engaging in change.^8^ Our study builds on existing research to show how clinical leaders are well placed to undertake these tasks.^9 11^ The findings support and extend Jones and Fulop’s^10^ study of board-level Medical Directors, specifically the idea that leaders bring insight and intelligence into decision-making, that they tactfully mediate conflict and rebuild relationships. More than thematic nomenclature, our study adds to the existing literature on clinical leadership in two ways. First, it further unpacks specific leadership activities, for example, those of critical challenge, maintaining the narrative, advocacy, alliance building and resource sharing. It also traces the connections between these activities and what we conceive of as, the linkages between primary and latent contributions. As outlined above, we show how activities related to representation and communication combine to provide a basis for shared vision and legitimacy.

Second, our study provides empirical evidence on the contributions of clinical leaders across multiple levels of health system governance, that is, the interorganisational level. It has been suggested that integrated care systems are illustrative of collaborative governance regimes^12^ in which multiple state and non-state actors participate in deliberative decision-making and consensus-building.^14^ As with other cases of network governance,^18^ the governance of STPs was also characterised by management hierarchy in which tiers of committee-like decision-making were located with vertical structures of delegation and accountability. Furthermore, involvement in thematic and project level committees was, at this time, based on informal cooperation and where clinical leaders engaged in representational advocacy and ‘shuttle diplomacy’.^14^ In some respects, this echoes the types of multitier distributed leadership identified by Fitzgerald *et al*^9^ but at the inter-organisational level.

Extending this line of discussion, our study offers insight into clinical leadership across different periods of system integration and tiers of system governance. With regards to the stages of STP development, clinical leaders’ contribution to early strategy setting seemed to centre on providing analytical insight, representing stakeholders’ interests and then translating and communicating strategies favourably to stakeholders. In later stages of collaborative working clinical leaders contributed to sustaining commitment and relationships through relational activities. In terms of governance tiers, leaders’ contributions varied at senior, middle and local levels, from a relatively small number of very senior clinical leaders providing high-level analytical insight and representational legitimacy, to specialist clinical leaders overseeing and coordinating thematic programmes by translating strategy and engagement stakeholders, and service-level clinical leaders coordinating integration projects with delegated authority and by sharing resources.^19^ Such temporal and positional variations in system governance are worthy of future research.

Our study gives further weight to the use of leadership theory for informing research and practice, while also suggesting that sensitively combining insights from multiple perspectives, rather than reliance on any one framework, may be appropriate given the contingencies in system governance. In different ways our findings reflect elements of ‘network’, ‘facilitative’ and ‘system’ leadership. With reference to McGuire’s^13^ work on network leaders, clinical leaders fostered and sustained commitment by, for example, representing viewpoints and communicating plans, they also supported integrative working and resource sharing through relational activities, and at the thematic and project levels engaged stakeholders in change initiatives. Clinical leaders’ activities reflected many of the aspects of facilitative leadership summarised by Ansell and Gash^14 15^ and, building on their subsequent work, many could be interpreted as ‘mediators’ given their role in resolving disputes and fostering shared understanding, and also ‘catalysts’ for engendering involvement in change. With reference to Senge *et al*’s^16^ notion of system leadership, clinical leaders were well positioned to ‘see the big picture’, foster shared understanding, engender reflexive conversations and promote proactive change through their translational and communication activities.^16^ In many regards these perspectives overlap and complement each other, but each emphasises different aspects of clinical leaders’ contribution to integrated care systems. We summarise these in terms of the structural (membership, rules, accountabilities), the procedural (decision-making, relationships, resource sharing) and the ideational (vision, values). Given the scale and heterogeneity of regional systems of integration, it may be expected that clinical leaders will vary considerably in how their activities are weighted against these domains.

### Limitations and future research

The study draws on a relatively small number of interviews from three STPs. There is scope to broaden and extend this line of research through carrying out additional forms of qualitative research and, based on the model outlined above, carrying out survey research with a larger, more diverse sample of clinical leaders. There is also scope to focus on particular sections of clinical leadership, for example, senior medical director role, or to compare between clinical leaders in terms of their professional background. It is also the case that STPs have evolved into newly formed ICSs which have modified governance arrangements with additional expectations for clinical leadership. As such, the research could be further updated in light of this policy change.

## Conclusions

Clinical leaders can make a distinct and significant contribution to the leadership of integrated care systems. These are shown across four interconnected sets of activities that make analytical, representational, translational and communication, and relational contributions, which help to construct a realistic and shared vision of integrated working, legitimise the proposed models of integration, promote engage and commitment to integration and facilitate collaboration and the sharing of resources. These contributions also vary according to where leaders are positioned in the governance structure and at the stage in the integration initiative. Further consideration to how these capabilities can be recognised and valued in integrated care systems, and how they can be developed among current and future system leaders is a priority given the continued significance of health and social care integration.

## Supplementary material

10.1136/leader-2022-000709 online supplemental file 1

## Data Availability

Data are available upon reasonable request.
