# Supplementary material for: What can clinical leaders contribute to the governance of integrated care systems?
Source: BMJ Lead. 2023 Mar 8;7(4):e000709. doi: 10.1136/leader-2022-000709 (PMC12038146; doi:10.1136/leader-2022-000709)
Supplement: online supplemental file 1 [file leader-7-4-s001.pdf]

Supplementary File

Table 2. Illustrative data and analysis

Illustrative data	Coding	Theme
I think the relationships within our system are very, very difficult actually… For me, it would look like somebody or a group of people who genuinely looked at the data, genuinely asked the questions and listened to the answers, genuinely stopped acting in a way that was self-protectionist and were prepared to lose some things to the greater value of the system. I think you'd have to start doing that as an example to others. (Site B: P3)<br><br>the only thing I can do is be very clear about the numbers, and then, from now on, I keep … I will develop very, very clear charts and graphs because I think she might understand a picture, and just keep, 'this is what we're doing, this is what we're doing, this is what we're doing. (Site C: P2)	Reviewing and presenting evidence	Analytical contribution
There is a long history of disagreement between those services and it's not just about money but it's about who has the beds or does the work, and what you are proposing is going to make this much worse (Site B: P2 field observation)	Underlying tensions	

So many departments, teams, organisations and the conflicts between them, in terms of understanding what the whole system needs but knowing what the regulatory landscape requires of them, within their particular silo, or within their particular department, or unit, or whatever it was. (Site C: P1)		
I think you need to have credibility that the people who are making the changes in the pathways are doing so with an understanding of the patient bases and the different sorts of patient bases that are populations that are in the area really (Site B: P5)<br><br>What I really want to make give the team is a really clear sense of what we can achieve. It has to be things we can actual deliver so not trying to do too much or massively transform roles, but more gradual (Site C: P1 field interview)	Determining feasible and relevant strategies	
Yes, and then you can help the policy makers and the people writing the plan see how it's going to make a difference to positive effect rather than just doing it because you have to? …We worked on developing a strategy as a team that got fed up to the STP board. Maybe with a lot of things it wasn't necessarily driven by the leadership board and I think at the end of the year that's what I was feeding back, our priorities need to be driven by what the priorities are. Again, you're trying to work with the people that get it and see how it could help them. (Site B: P4)	Ensuring clinical voice	Representational

You need to really listen to and understand the GPs perspective. If you push through your view of how we should organise primary care then the doctors will disengage, they won't be happy and in the long run they want play their full part in the system (Site B: field observation)		
In a way I'm a system leader at the senior level, I'm also a system leader internally, quite senior within the trust, and I'm also frontline go to the team, see the patients and do the MDT clinician. And there's such a mismatch in pace, the pace of life and the amount of time available. So, I think I'm at that point now I've got to go back in and say well, hang on all the teams are in a similar position, what we've got to offer is a twenty-minute thing not their four-hour thing and that's the way to engage and I don't think they realise that. … explaining that this is what's gone wrong (Site B: P1)<br><br>I would prefer to stay as individual organisations because the populations in the different areas are very different and I am concerned that the stronger, more politically minded area where the patients tend to be more educated and middle, and that concerned me that the service development will be aimed at the areas that speak loudest and less at the areas with high deprivation. (Site B: P4)	Critical challenge	
It's about being inclusive… so people can feel engaged and inspired….I know how difficult it can be to try to force people, doctors, to accept change and it has feel that it is driven by the community (Site C: P4 field interview)	Engagement	

I will be really measured and careful with the use of language and with how I write it to maintain and retain engagement. I work really hard not to disengage, not to cause disengagement because as soon as you start to lose people, you've lost something big haven't you? [Site B: P1]		
Now data is never going to be perfect is it, but we have a lot of data, we have a lot of facts and figures all over the place. The ability to analyse that into proper information is really important. The ability to then have a conversation and all trust that, because it's all we've got and we put that together with patient experience, information with professional body information, with guidelines, with evidence-based practice, with research evidence and pull it altogether. (Site B: P3)<br><br>If it doesn't make sense to people they won't buy into it, and much of the system change framework seems so detached from what is going on locally, you know, it's quite abstract. So, I help to make it make sense by showing what it can actually change things for the better. (Site C: field interview)	Translation of strategy	Translation and communication
As long as you link what you want to do…"it's a really good idea because it's all about collaboration"…or "this will help them deliver the Long Term Plan" so you have to link what you want to do to one of the key areas". (Site A: P1)	Favourable framing	

you can talk about how, well if we can work better together as a group and you may lose a little bit and we may lose a little bit. We work together and in an altruistic way it's better for patients, it might be more efficient, some losers, some gainers, that kind of approach, give and take approach, and our negotiations can occur in that fashion. (Site B, P2)		
They need to keep hearing about the successes, what its changing for the better. Show them that these projects do make a difference and that when you add them all up we are seeing something bigger going on. It like I said, about showing people the big picture, but also show them how the bit fit together in that (Site B: field interview)	Keeping the story going	
So developing, some of it’s been about developing relationships and alliances, …, getting the, a better clearer shared understanding across the different stakeholders about the current situation and what the facts are and what the evidence is, and what needs to change.<br>(Site A: P5)<br><br>....give them the head space to think about what they needed to do and coming together as a group and really working … deciding what they were going to do and [Name] having time to put things together and talk to the people they needed to talk to in the wider STP footprint etc. (Site A: P1)	Brokering and building connections	Relational

Really, you're getting lots of different people at different levels to influence the system, it's building your tribe of champions to share and we like the film of the man dancing on the hill where you're growing your tribe. We definitely build a much wider spread, or found people that already wanted to work like that. (Site B: P4)	Self-sustaining networks	
it's a different way that you lead, you have to, it's facilitating and coaching, not this is how you're going to do it and everybody's going to do it the same…showcasing the small seeds of innovation tells people what's in it for them (Site B: P1)<br><br>encouraging others to take the lead ensuring what happens is right rather than who decides what happens. So it's terribly important that the right thing happens rather than them looking to the boss (Site C: P2)<br><br>There are obviously a lot of financial and clinical issues that we cannot bottom-out today, so can I ask that you review these and prepare something our next meeting (Site B: P3 fieldnote)	Empowering and delegating	
It's so important to find the things that will keep them interested, it doesn't always have to be more resource or pandering to their egos, it is better when it's about the issues that really matter to the service and communities. (Site B: P4)	Mediating conflict	

People are a valuable asset and people are under a lot of stress and not helped if you're in an environment where you've got all the conflict and problem. I think you learn, you look for the opportunities and shared agendas. (Site B: P1)<br><br>[T]here's an element of how to create win-win situations because if you go into something and there's clearly going to be a loser there that's not going to, depending on them and how they're going to manage it (Site A: P2)		
